# Phenotypic and genomic characterization of azole resistance in Portuguese *Candida parapsilosis* isolates

**DOI:** 10.3389/ffunb.2025.1713961

**Published:** 2025-12-10

**Authors:** Alexandru-Marian Papuc, Cristina Veríssimo, Helena Simões, Cristina Toscano, João Paulo Gomes, Verónica Mixão, Raquel Sabino

**Affiliations:** 1 Reference Unit for Parasitic and Fungal Infections, Department of Infectious Diseases, National Institute of Health Doutor Ricardo Jorge (INSA), Lisbon, Portugal; 2 Microbiology Laboratory, Unidade Local de Saúde Lisboa Ocidental, Hospital Egas Moniz, Lisbon, Portugal; 3 Genomics and Bioinformatics Unit, Department of Infectious Diseases, National Institute of Health Doutor Ricardo Jorge (INSA), Lisbon, Portugal; 4 Animal and Veterinary Research Center (CECAV), Faculty of Veterinary Medicine, Lusófona University - Lisbon University Centre, Lisbon, Portugal; 5 Research Institute for Medicines (iMed.ULisboa), Faculdade de Farmácia, Universidade de Lisboa, Lisbon, Portugal; 6 Instituto de Saúde Ambiental, Faculdade de Medicina, Universidade de Lisboa, Lisbon, Portugal; 7 Laboratório Associado TERRA, Instituto Superior de Agronomia, Lisbon, Portugal

**Keywords:** *Candida parapsilosis*, resistance, fluconazole, ERG11, whole-genome sequencing

## Abstract

**Introduction:**

*Candida parapsilosis* is a clinically important etiological agent of systemic infections associated with hospital outbreaks, which prevalence has been increasing in the last decade. Moreover, in recent years, fluconazole resistance in this species has been emerging in different countries, being a subject of significant interest and concern. In this context, the present study aims to determine the frequency of fluconazole resistance in *C. parapsilosis* sensu stricto isolates collected in Portugal (2003–2007 and 2017-2024), understand its associated molecular mechanisms, and relate it all with worldwide genomic data.

**Methods:**

To this end, we performed phenotypic assays of 145 isolates of *C. parapsilosis* collected from different biological and environmental products in Portugal (majority from the Lisbon Metropolitan area), and explored the genomic features of the fluconazole-resistant ones.

**Results:**

We found eight *C. parapsilosis* fluconazole-resistant isolates between 2017 and 2024, corresponding to a frequency of 8.5% in this period, and contrasting with the absence of fluconazole-resistant isolates collected before 2007. Sequencing of the *ERG11* gene showed that all fluconazole-resistant isolates had the Y132F and R398I mutations.

**Discussion:**

A phylogenomic analysis including publicly available isolates from other countries revealed that our Portuguese isolates are more closely related to those from the USA and Germany than to the isolates sequenced thus far from the neighbor country, Spain. Furthermore, although three distinct *C. parapsilosis* genetic clades were found in our dataset, all the fluconazole-resistant isolates detected in this study cluster together, raising the question of whether the increased fluconazole-resistance in the country could possibly be associated with the emergence or introduction of this particular lineage. Altogether, these results provide valuable insights on fluconazole resistance in a set of Portuguese *C. parapsilosis* isolates and their associated mechanisms, representing an important step towards a better understanding of the increasing *C. parapsilosis* fluconazole resistance in Southern Europe.

## 1 Introduction

*Candida parapsilosis* is a common element of the microbiota of the skin, gastrointestinal tract, and mucous membranes of the genital and oral cavities (Heisel et al., 2015; Pappas et al., 2018; Liu et al., 2022; Kreulen et al., 2023; Nascimento et al., 2025). *Candida parapsilosis* species complex includes, besides *C. parapsilosis* sensu stricto, *Candida orthopsilosis* and *Candida metapsilosis*, two species that until 2005 were considered different *C. parapsilosis* groups (Tavanti et al., 2005), as well as *Candida theae* (Chang et al., 2012) and *Candida margitis* (Liu et al., 2016), which were recently assigned to this species complex based on genome-scale analysis (Mixão et al., 2022; Gabaldón, 2024). Except for *C. margitis*, all these species are able to cause human disease, with *C. parapsilosis* s.s. being the one causing the highest number of infections (Silva et al., 2009; Gonçalves et al., 2010; Arendrup et al., 2023; Gabaldón, 2024). As such, the majority of studies on epidemiology and susceptibility focus specifically on this species (Escribano and Guinea, 2022; Rodrigues et al., 2022; Asogan et al., 2024). The highest incidence of infection caused by this species occurs among individuals with impaired immunity, newborn infants, those undergoing chemotherapy, and patients in intensive care units (Franconi et al., 2023). Nevertheless, over the past two decades, its prevalence has increased significantly, rendering it one of the most prevalent fungal agents of nosocomial infections (Arendrup et al., 2023), being no longer limited to a specific patient population, as immunocompromised or neonates (Hubler et al., 2023). This species exhibits essential virulence factors that contribute to the growing success of the infections it causes. These include adhesion to host cells, biofilm formation, and secretion of hydrolytic enzymes such as proteases, phospholipases, and lipases (Trofa et al., 2008). The formation of biofilms gives the organism a greater affinity for medical devices and increased resistance to antifungal drugs (Branco et al., 2023).

According to the global 2025 ECMM/ISHAM/ASM *Candida* guidelines (Cornely et al, 2025), echinocandins are strongly recommended as first-line treatment of candidaemia. If echinocandins are unavailable or the patient is colonized or was previously infected with echinocandin-resistant strains, liposomal amphotericin, fluconazole and voriconazole are moderately recommended. Although members of the *C. parapsilosis* species complex have higher echinocandin Minimum Inhibitory Concentrations (MICs) owing to a naturally occurring polymorphism in *FKS1* gene, clinical response to this drug appears similar to other *Candida* species. Still, in many low-income and middle-income countries, fluconazole is the most widely used antifungal for treating *Candida* infections, as alternative treatments are costly or not readily available. Additionally, fluconazole is highly recommended for *Candida* infection prophylaxis in different groups of patients (Lass-Flörl, 2025). However, this guideline also underlines the importance of antifungal stewardship, source control, and tailored treatment approaches based on patient-specific risk factors and local epidemiological data. As such, knowing the epidemiology and resistance rate of *C. parapsilosis* in a specific local/country is essential to choose the prophylactic antifungal, namely fluconazole (Cornely et al., 2025; Lass-Flörl, 2025).

Due to its frequent use in treating these infections, resistance to fluconazole has emerged among *C. parapsilosis* isolates (Daneshnia et al., 2023). Several molecular mechanisms are associated with fluconazole resistance in *Candida* spp., including point mutations in the ergosterol biosynthesis pathway (*ERG3*) that allow growth in the presence of azoles, or the overexpression of multidrug efflux pumps, namely *CDR1* and *CDR2*, as well as the *MDR1* genes (Branco et al., 2023). The *CDR1* and *CDR2* are ATP-binding cassette (ABC) transporters, while the *MDR1* is a major facilitator superfamily (MFS) (Doorley et al., 2022). These transporters are crucial for yeast survival under stress to antifungal or other harmful components, extruding a wide range of potentially toxic substances from the cytoplasm to the outside environment, protecting the cell. Thus, beyond drug resistance, these transporters help yeast in case of environmental stress, including the presence of toxic compounds such as alcohols, weak acids, and heavy metals. They are also involved in regulating cell metabolism as in ion transport, transmembrane transport, and carbohydrate metabolism, playing an important role in membrane homeostasis as well (Bereketoglu et al., 2017; Kumari et al., 2021; Branco et al., 2023). However, the principal resistance mechanism to fluconazole in *C. parapsilosis* are mutations in the *ERG11* gene, which result in the over-expression of the enzyme lanosterol 14-alfa-desmethylase (Erg11p) and in the reduction or loss of affinity of the azoles to this enzyme. Indeed, the most prevalent amino acid substitution associated with fluconazole resistance in *C. parapsilosis* is Y132F in *ERG11* (Castanheira et al., 2020), which involves the replacement of tyrosine with phenylalanine at position 132. This substitution has been so far also documented in *C. albicans*, *C. tropicalis*, and *C. auris* (Escribano and Guinea, 2022).

In 2022, the World Health Organization (WHO) classified *C. parapsilosis* as of high-risk level of threat, due to its increasing prevalence and the emergence of antifungal resistance (World Health Organization, 2022), which is particularly evident in Southern Europe (Castanheira et al., 2020; Arendrup et al., 2023). For instance, a study conducted in 2023 by Díaz-García et al. (2023), involving *Candida* isolates collected in Madrid hospitals, demonstrated an increase of candidemia cases caused by *C. parapsilosis*, along with a corresponding rise in fluconazole-resistant isolates (Díaz-García et al., 2023). Particularly, the city of Madrid has seen a dramatic rise in the occurrence of *C. parapsilosis* isolates resistant to fluconazole (collected from blood cultures and intra-abdominal samples), from 2.6% in 2019 to 36.6% by 2023 (Díaz-García et al., 2023). In other countries and continents, this concern can be even greater due to the upward trend of invasive candidiasis caused by *C. parapsilosis* over the past decade, with an associated mortality rate of between 20% and 45% (World Health Organization, 2022). In 2024, and under the WHO report, a group of scientists highlighted the emergent relevance of *C. parapsilosis* and the urgent need for ongoing research and surveillance worldwide (Asogan et al., 2024).

Some studies have been conducted with isolates collected from different biological products to clarify the incidence of *C. prapsilosis* and its resistance to fluconazole in Portugal (Silva et al., 2009; Faria-Ramos et al., 2014; Pinto-Magalhães et al., 2019; Nascimento et al., 2024). For instance, the most recent study, conducted between 2020 and 2022, found that two out of the 89 *C. parapsilosis* complex isolates collected from the axilla/groin (by bilateral swabs) from ICU patients were resistant to fluconazole (Nascimento et al., 2024). Nevertheless, to the best of our knowledge, the genetic traits potentially associated with resistance have not yet been explored. Given the significant role of *C. parapsilosis* as a causative agent of systemic infections and hospital outbreaks in Portugal (Branco et al., 2023), the knowledge of the azole resistance rate and molecular mechanisms associated with resistance became an unmet need. Therefore, this study aims to determine the frequency of fluconazole resistance in a set of *C. parapsilosis* isolates from the collection of the Portuguese National Reference Laboratory, collected from different biological products, and to identify the molecular mechanisms potentially associated with it in the largest Portuguese collection published to date. Finally, we also compared the genomic data of the identified fluconazole-resistant isolates under study with isolates from other geographical origins to perceive their genetic relatedness.

## 2 Materials and methods

### 2.1 Collection, selection, and identification of yeast isolates

All *C. parapsilosis* s.s. isolates stored in the collection of the Portuguese Mycology National Reference laboratory between 2017 and 2024 (N = 94) were analyzed in this study, with the majority being prospectively isolated in the period between 2023 and 2024. In a second phase of the work, 51 other *C. parapsilosis* s.s. isolates, collected in the period 2003-2007, were added to the study for comparison (**Supplementary File 1**). These isolates were selected from a database of more than 200 *Candida* isolates from the same period available at the Mycology National Reference Laboratory. All isolates from blood cultures and hospital environment from this collection were selected based on the higher likelihood that strains isolated from cases of candidemia or from environmental settings had been exposed to clinical antifungals or disinfectants, thereby increasing the potential for antifungal resistance development.

The identification of these isolates as *C. parapsilosis* s.s. was performed either by microsatellite genotyping (isolates 2003-2007) (Sabino et al., 2010) or with matrix-assisted laser desorption ionization-time of flight mass spectrometry (MALDI-TOF MS) (isolates >2017); identification of all was double checked by ITS sequencing. The identification by MALDI-TOF MS was set up according to the manufacturer instructions (Bruker Daltonics, Bremen, Germany, 2023). The ITS1/4 pair of primers used were ITS1 – 5’ TCCGTAGGTGAACCTGCGG 3’ and ITS4 – 5’ TCCTCCGCTTATTGATATGC 3’. The amplification of the ITS1/4 region was set up with puReTaq Ready-To-Go PCR Beads following the manufacturer’s instructions (Cytiva, Marlborough, MA, USA). PCR cycles were one cycle of initial denaturation at 95°C for 4 min 30 sec, 40 cycles of denaturation at 95°C for 30 sec, annealing temperature at 50°C for 30 sec, extension at 72°C for 1 min, and one cycle of final extension at 72°C for 3 min in ThermoFisher Thermal cycler. PCR products were analyzed by 2% agarose gel. PCR products generated for the ITS1/4 region were purified using the illustra™ ExoProStar™, GE Healthcare Life Sciences (Buckinghamshire, UK), before DNA sequencing. Sequencing of both strands was performed with the BigDye terminator v 1.1 cycle sequencing kit (Applied Biosystems) using the same primers. Sequencing conditions were as follows: an initial denaturation at 96°C for 5 sec, followed by 30 cycles of 96°C for 10 sec, 50°C for 5 sec, and 60°C for 4 min, followed by one cycle of 72°C for 5 min (Sabino et al., 2014). The obtained ITS sequences were compared with sequences from the NCBI database (https://www.ncbi.nlm.nih.gov), using the Basic Local Alignment Search Tool (BLAST) (https://blast.ncbi.nlm.nih.gov/Blast.cgi). The identification of *C. parapsilosis* isolates was contingent upon two criteria: (1) a minimum of 98% identity and (2) a minimum of 98% coverage, with no background noise.

### 2.2 Susceptibility testing

The susceptibility testing was done using agar screening plates and by the EUCAST microdilution methods. The screening tests were carried out by using Sabouraud dextrose agar (SDA) plates without chloramphenicol supplemented with fluconazole at a concentration of 8 mg/L, since according to the EUCAST guidelines, the clinical cut-off value is a MIC >4 mg/L (to classify *C. parapsilosis* as resistant to fluconazole). To control strain viability, an SDA plate without chloramphenicol was used.

Fresh yeast cultures grown on SDA plates were suspended in saline solution at a turbidity equivalent to a 0.5 McFarland standard. Plates were inoculated by swabbing and incubated at 37°C for 24 h. Four isolates were employed as controls, comprising two fluconazole-resistant [*Candida krusei* (*Pichia kudriavzevii*) ATCC 6258 and *Candida parapsilosis* NQ6714] and two fluconazole-susceptible isolates (*Candida albicans* ATCC 10231 and *Candida parapsilosis* ATCC 22019). In case of growth in the agar media supplemented with fluconazole, the isolates were classified as potentially resistant and considered for further confirmatory tests with broth microdilution assays.

### 2.3 Broth microdilutions assay

Microdilutions method was carried out using the MICRONAUT system (Bruker, Massachusetts, USA), according to the manufacturer’s instructions. Briefly, a yeast suspension of 0.5 McFarland was prepared in NaCl 0.9%. The suspension was then diluted 1:1000 into 11.5 mL of MICRONAUT-RPMI-1640 medium (containing 4% of glucose and MOPS). Then, 100 µL of working solution containing 1–5 × 10^3^ CFU/mL was distributed into each well with a multi-channel pipette. *Candida parapsilosis* ATCC 22019, *C. parapsilosis* PNAEQ 6714, and *C. krusei* ATCC 6258, were used as quality controls. The plates were incubated at 37°C. They were read visually under normal laboratory lighting. After 24 h of incubation, the growth control well was examined. When the growth was evident, the MIC values for the antifungal agents were read out, in contrast, the test plates were re-incubated and re-examined after 48 h. The MIC was read according to the recommendations of the manufacturers as the lowest concentration of each antifungal where no growth was observed.

### 2.4 Sequencing of *ERG11* for detection of mutations

In a first step, we aimed to quickly check for mutations in the *ERG11* gene. With that purpose, we performed a PCR targeting that gene, followed by sequencing. The DNA was extracted from each isolate using the High Pure PCR Template Preparation Kit from Roche Life Science (Basel, Switzerland). The *ERG11* gene was amplified by PCR using the primer pairs designed by Grossman et al. (2015). Amplification was set up with puReTaq Ready-To-Go PCR Beads (GE Healthcare, Buckinghamshire, United Kingdom) with a total reaction volume of 25 μL that contained 12 μL of H_2_O, 5 μL of extracted template DNA, and 0.4 μmol (4 μL) of each forward and reverse primer. Reactions were run with an initial denaturing step at 94°C for 5 min, followed by 35 cycles of 94°C for 30 sec, 58°C for 30 sec, and 72°C for 1 min, followed by final extension at 72°C for 7 min. Forward and reverse sequencing reactions were performed under the conditions previously described for ITS sequencing. Of note, due to methodological constraints, isolate CpPT17069 was not subjected to conventional PCR directed at the *ERG11* gene.

### 2.5 Whole-genome sequencing

In a second step of our study, we performed WGS to the selected isolates. With this methodology we aimed to confirm the previous data obtained by Sanger sequencing and further explore: i) the genetic diversity within the fluconazole-resistant isolates analyzed in this study; ii) the presence of additional mutations of interest in these isolates; and iii) the existence of genomic factors potentially associated with the intermediate phenotypes observed. Hence, for whole-genome analysis, total DNA from the *C. parapsilosis* isolates with an MIC ≥4 mg/L (including isolate CpPT17069, N = 11) was subjected to Nextera XT library preparation and paired-end sequenced on an Illumina NextSeq 2000, according to the manufacturer’s instructions, at the Technology and Innovation Unit of the Portuguese National Institute of Health (INSA). Sequencing reads were inspected with FastQC v0.11.9 (Andrews, 2010) and filtered with Trimmomatic v0.39 (Bolger et al., 2014). The *K-*mer Analysis Toolkit (KAT) was used to count the 27*-*mer (default) frequency and GC content and to estimate the expected genome size (Mapleson et al., 2017). To analyze the sequencing data, a read mapping strategy was employed on the *C. parapsilosis* reference genome (NCBI accession: GCF_000182765.1) using BWA MEM 0.7.17-r1188 (Li, 2013). The GATK program, version 4.2.6.1 (McKenna et al., 2010), was employed to sort the alignments by coordinate (gatk SortSam), mark duplicates (gatk MarkDuplicates), generate BAM index (gatk BuildBamIndex) and obtain alignment metrics (gatk CollectAlignmentSummaryMetrics). This tool was also used to identify SNPs and INDELs using HaplotypeCaller algorithm for a ploidy of 2, and to filter these variants with VariantFiltration tool, with a minimum coverage of 30 reads being considered (‘-filter DP <= 30’). Except when specified otherwise, all bioinformatics tools were run with default parameters. The Integrative Genomics Viewer (IGV) software, version 2.18.0 (Robinson et al., 2023), was employed to visualize the complete set of alignments. It should be noted that the reference genome strain (CDC 317) has a fluconazole-resistant phenotype (Bergin et al., 2022). Of note, at a later stage, seven susceptible isolates were also subjected to WGS and included in this analysis to complement the data on isolates from superficial infections, since only one from this source was initially analyzed (CpPT17069). Hence, isolates from nails (CpPT17039, CpPT17040, CpPT17046, CpPT17048 and CpPT17050) and vaginal exudates (CpPT17041 and CpPT17045) were randomly selected and further added.

### 2.6 Phylogenetic analysis of the isolates

In order to assess the genetic relatedness of the isolates sequenced in this study with those from alternative sources and geographical locations, we retrieved from the NCBI Sequence Read Archive (SRA) the Illumina raw sequencing data of the 51 isolates used in a previous study relating genomic patterns and drug resistance in *Candida* spp (Schikora-Tamarit and Gabaldón, 2024). and of the 15 sequenced isolates from Spain available until the date of this study (Guinea et al., 2021). These genomes were subjected to the same read-mapping methodology that was employed for the samples sequenced in the frame of this study. The same methodology described by (Mixão et al., 2023) was employed to reconstruct the phylogenetic tree. In summary, for each sample, homozygous variants were replaced in the reference genome (*Candida parapsilosis* CDC 317; NCBI accession: GCF_000182765.1) with GATK version 4.2.6.1 (McKenna et al., 2010), using the FastaReferenceMaker module. All positions with heterozygous variants or insertions/deletions in at least one sample were removed from the alignment using bedtools version 2.31.1 (Quinlan and Hall, 2010), resulting in a final alignment comprising 12954955 nucleotides with 8394 variable sites. The maximum likelihood tree reconstruction of this alignment was generated with RAxML version 8.2.8 using the GTRCAT model (Stamatakis, 2014). The tree was rooted using the midpoint rooting method.

### 2.7 Statistical analysis

A Fisher’s exact test (Raymond and Rousset, 1995) was employed and conducted in RStudio (Version 2023.03.0 + 386), whereby a contingency table (2x2) was initially generated, with the number of resistant/non-resistant cases and the date at which the sample was obtained or the countries of origin, as well as the prevalence of the Y132F or Y132F + R398I mutation between resistant isolates in different countries (Horton and Kleinman, 2015). The significance level was set at 0.05. Whenever the *p*-value was less than 0.05, the null hypothesis was rejected, and a significant difference between the data tested was detected.

## 3 Results

### 3.1 Fluconazole resistance in the Portuguese *C. parapsilosis* isolates

**Figure 1:**
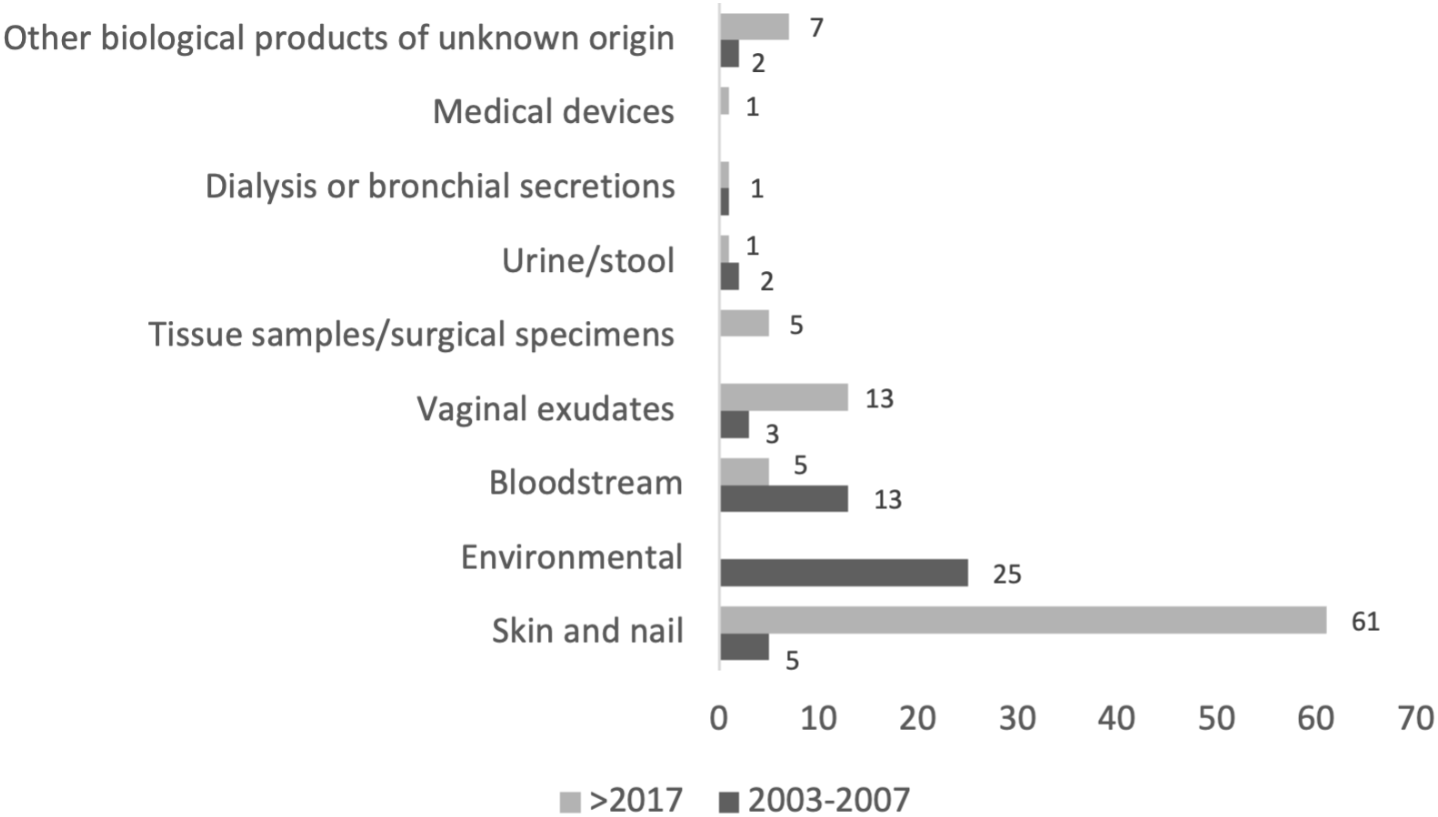
Distribution of isolates by origin and collection period.

The 145 isolates were obtained from different biological sources, including skin and nails (N = 66; 46%), environmental samples (N = 25; 17%) [which include hospital surfaces (N = 22) and beach sand (N = 3)], bloodstream (N = 18; 12%), vaginal exudates (N = 16; 11%), and tissue samples/surgical specimens (N = 5; 3%). The remaining isolates were collected from urine/stool (N = 3; 2%), products from dialysis or bronchial secretions (N = 2; 1%), medical devices (N = 1; 1%), and other biological products of unknown origin (N = 9; 6%) (**Figure 1**; details in **Supplementary file 1**). The identification of *C. parapsilosis* s.s. was confirmed by ITS sequencing in all isolates.

Of the 94 *C. parapsilosis* isolates (2017-2024) subjected to screening, 23 showed possible resistance to fluconazole as they grew on the agar plates supplemented with this antifungal. To compare resistance rates across different time periods, additional 51 isolates were selected from the laboratory’s culture collection, including all isolates from blood cultures and hospital environment from the period 2003 to 2007. None of these isolates showed any resistance in the screening tests. From all isolates subjected to screening, only eight isolates from the batch 2017–2024 exhibited MICs ≥8 mg/L, corresponding to 5.5% (8/145) of the isolates classified as fluconazole resistant among the tested isolates and to 8.5% (8/94) of those collected between 2017 and 2024 (**Table 1**). As the remaining 15 isolates with demonstrated growth in the screening medium showed, by microdilution testing, MICs corresponding to the susceptible category (MIC ≤4 mg/L), they were considered as false positives of the initial screening and classified as susceptible. Our resistance rate from >2017 was compared with the resistance rate obtained by Faria-Ramos et al. (2014), and no statistically significant difference was found (*p*-value = 0.49), despite the considerable difference in the number of bloodstream isolates analyzed in each study.

**Table 1:** Source, collection year, minimal inhibitory concentration, and *ERG11* mutations of the isolates suspected to be resistant to fluconazole.

Isolate	Source	Collection year	MIC	*ERG11*
				Aminoacid change
CpPT17012	Thoracic Valve	2022	32	Y132F; I197I; R398I
CpPT17015	Isolate of unknown biological product from Cardio-Thoracic Surgery patient	2022	16	Y132F; I197I; R398I
CpPT17021	Isolate of unknown biological product	NA	64	Y132F; I197I; R398I
CpPT17049	Isolate of unknown biological product from Neurosurgery patient	2023	32	Y132F; I197I; R398I
CpPT17069	Right toenail	2023	4	NP
CpPT17138	Thoracic Valve	2017	4	I197I
CpPT17139	Blood culture	2022	32	Y132F; I197I; R398I
CpPT17140	Isolate of unknown biological product	2024	32	Y132F; I197I; R398I
CpPT17142	Blood culture	2018	128	Y132F; I197I; R398I
CpPT17143	Cardiac valve - Intensive Care Unit 2	2024	64	Y132F; I197I; R398I
CpPT17144	Cardiac valve - Intensive Care Unit 1	2024	4	I197I

NA, not available; NP, Not performed.

All isolates with MIC ≥4 mg/L were submitted for identification by sequencing the internal transcribed spacer (ITS) region of the ribosomal RNA gene and their identification as *C. parapsilosis* s.s. was confirmed. The characterization of the *ERG11* gene was conducted on those ten isolates, comprising eight resistant (MIC >4 mg/L) and two susceptible to fluconazole (MIC = 4 mg/L, for comparison), as reported in **Table 1**. All resistant isolates exhibited the mutations Y132F and R398I (**Tables 1**, **2**). This finding aligns with the established literature on the subject, as all isolates that exhibit the Y132F mutation have proven to be resistant to fluconazole (Hubler et al., 2023). All 10 isolates whose *ERG11* gene was sequenced exhibited a silent mutation I197I (T591C) (**Table 1**).

**Table 2:** Isolates subjected to WGS, their genetic clade, MICs to fluconazole and mutations identified in genes of interest.

Isolate	Genetic clade (Bergin et al., 2022)	Screening	MIC FLC (mg/L)	*ERG11*		*FKS1*		*MRR1*	*MDR1*	*CDR1*
				R398I	Y132F	D877N	R871G	D615G	I396V	Q935K
CpPT17138	4	+	4	–	–	–	–	–	–	–
CpPT17142	5	+	128	+	+	–	–	–	+	+
CpPT17139	5	+	32	+	+	–	–	–	+	+
CpPT17012	5	+	32	+	+	–	–	–	+	+
CpPT17015	5	+	16	+	+	–	–	–	+	+
CpPT17021	5	+	64	+	+	–	–	–	+	+
CpPT17041	1	–	NP	–	–	–	–	./+	+	–
CpPT17045	5	–	NP	+	–	+	–	–	–	–
CpPT17049	5	+	32	+	+	–	–	–	+	+
CpPT17050	5	–	NP	+	–	–	–	./+	+	–
CpPT17039	5	+	1	+	–	–	–	–	./+	–
CpPT17046	5	–	NP	+	–	–	–	–	./+	–
CpPT17040	5	–	NP	+	–	–	./+	–	–	–
CpPT17048	5	–	NP	+	–	–	–	–	./+	–
CpPT17069	5	+	4	+	–	–	–	–	–	–
CpPT17140	5	+	32	+	+	–	–	–	+	+
CpPT17143	5	+	64	+	+	–	–	–	+	–

This table only includes genes from the defined list of targets where mutations have been found.

The reference genome was *C. parapsilosis* CDC 317 (NCBI accession: GCF_000182765.1), a fluconazole-resistant isolate. (-) the mutation was not observed; (+) the mutation was observed; (./+) – the mutation is heterozygous; NP, not performed.

### 3.2 Genomic variability of the fluconazole-resistant isolates

Aiming to get further insight into the genomic variability of the *C. parapsilosis* isolates analyzed in this study and explore genomic factors potentially associated with the intermediate phenotypes observed, given the impossibility of sequencing all of them, we prioritized all isolates with an MIC ≥4 mg/L for WGS and comparative genomics analysis (**Table 2**; **Supplementary File 1**). As a preliminary analysis revealed that isolate CpPT17069 (isolated from a nail) presented interesting genomic patterns (details presented below), we also sequenced seven fluconazole susceptible isolates from superficial infections in order to explore the possibility that such patterns could be related with tropism (**Supplementary File 1**). An initial quality control of the sequencing reads (details in the Materials and methods section) revealed contamination in one of the fluconazole-susceptible isolates (CpPT17144), and, for this reason, it was excluded from further analysis. The remaining isolates were then subjected to a comprehensive genomic analysis with a special focus on genes with a potential role in antifungal resistance and virulence, including the *ERG11*, which had already been sequenced using Sanger methods, and *ERG3*, *MRR1* and *MDR1*, *CDR1* and *CDR2* (Tóth et al., 2019). Despite not being involved in fluconazole resistance, *FKS1* was also considered as a gene of interest to explore due to its particular relevance for *C. parapsilosis* antifungal resistance profile (Gabaldón, 2024). All non-synonymous SNPs found in these genes are detailed in **Table 2**. We highlight the finding of two non-synonymous mutations in the fluconazole-resistant isolates (besides the Y132F in *ERG11*), which are the I396V in the *MDR1* gene and the Q935K in the *CDR1* gene. While the first was present in all fluconazole-resistant and some fluconazole-susceptible isolates, the later was exclusive of the fluconazole-resistant isolates being present in all but CpPT17143.

**Figure 2:**
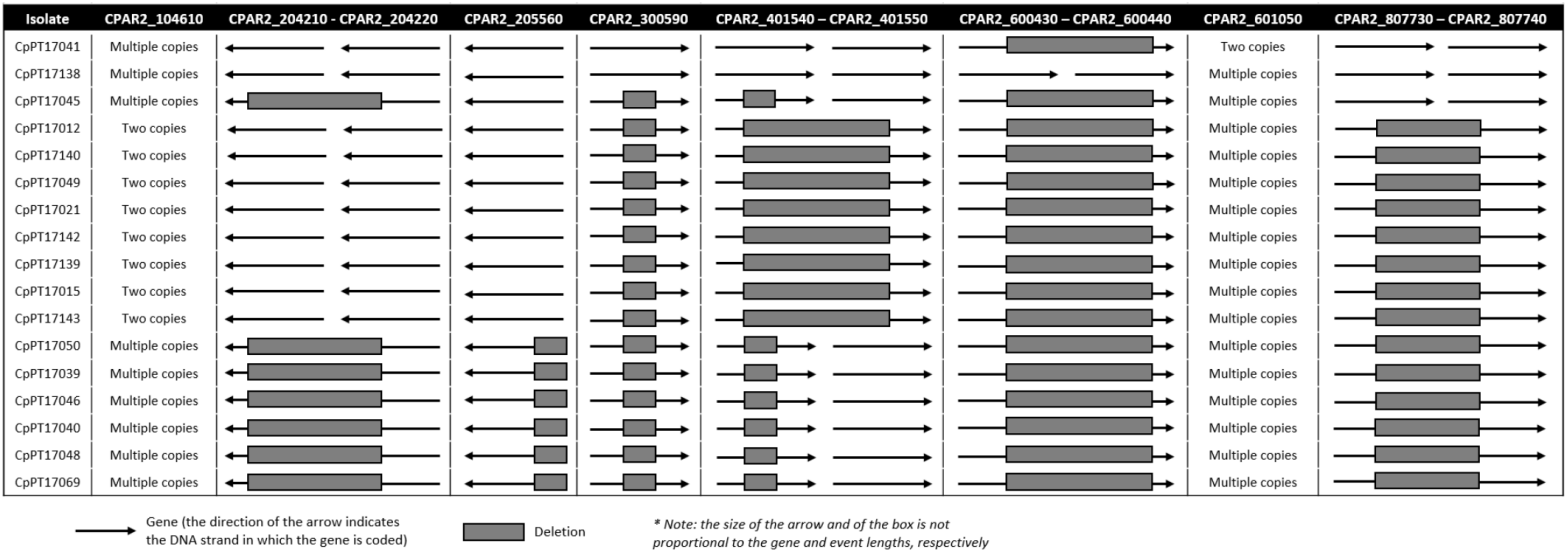
Genomic events detected through WGS analysis (gene deletions and copy number variations), with indication of the genes where such events occurred. All events are presented in comparison to the reference genome (NCBI accession: GCF_000182765.1). The order of the isolates reflects their position in the phylogenetic tree presented in **Figure 3**.

A further inspection of these genomes revealed gene deletions/copy number variations (CNVs) in these isolates (**Figure 2**), with CpPT17069 (intermediate phenotype) exhibiting distinctive CNV patterns that warranted further investigation. Specifically, this isolate presented a CNV in each and every gene where at least one of the other isolates of the initial dataset also presented this type of variability (not always with the same genomic coordinates). This isolate also had the particularity of being the only one isolated from a superficial infection. Therefore, to assess whether its CNV patterns could be related to tropism, susceptible isolates from a similar source were further sequenced and analyzed for comparison. The CNVs found in the analyzed dataset are detailed in **Figure 2**. Genes CPAR2_104610 (*RTA3*), implicated in antifungal drug resistance (West et al., 2021), and CPAR2_601050 (*ARR3*), involved in transmembrane transport activity, presented more than two copies in several isolates. However, in CPAR2_104610, this type of variability was not observed in the fluconazole-resistant isolates analyzed in this study. Also, some isolates presented deletions overlapping genes involved in cellular processes associated with fitness or structural components (CPAR2_401550//CPAR2_401540; CPAR2_807730//CPAR2_807740; CPAR2_300590) (Skrzypek et al., 2017), or genes (CPAR2_204210 and CPAR2_204220) which deletion results in a reduced growth rate in alkane-supplemented media (Panwar et al., 2001; Bergin et al., 2024). Interestingly, all five isolates collected from nail samples that were added at a later stage for comparison with isolate CpPT17069 revealed the same deletions/CNVs patterns as this isolate (**Figure 2**, **Supplementary File 1**). Moreover, we also detected a recombination between chromosomes HE605206 and HE605207 in isolates CpPT17039, CpPT17040, CpPT17046, CpPT17048 and CpPT17069, all of them originated from this source. Noteworthy, the comparison of the two isolates with intermediate phenotype between them and with the fluconazole-resistant ones did not reveal any trait that, in light of current scientific knowledge, could be suggested as potentially associated to a MIC of 4 mg/L.

### 3.3 Phylogenetic analysis

**Figure 3:**
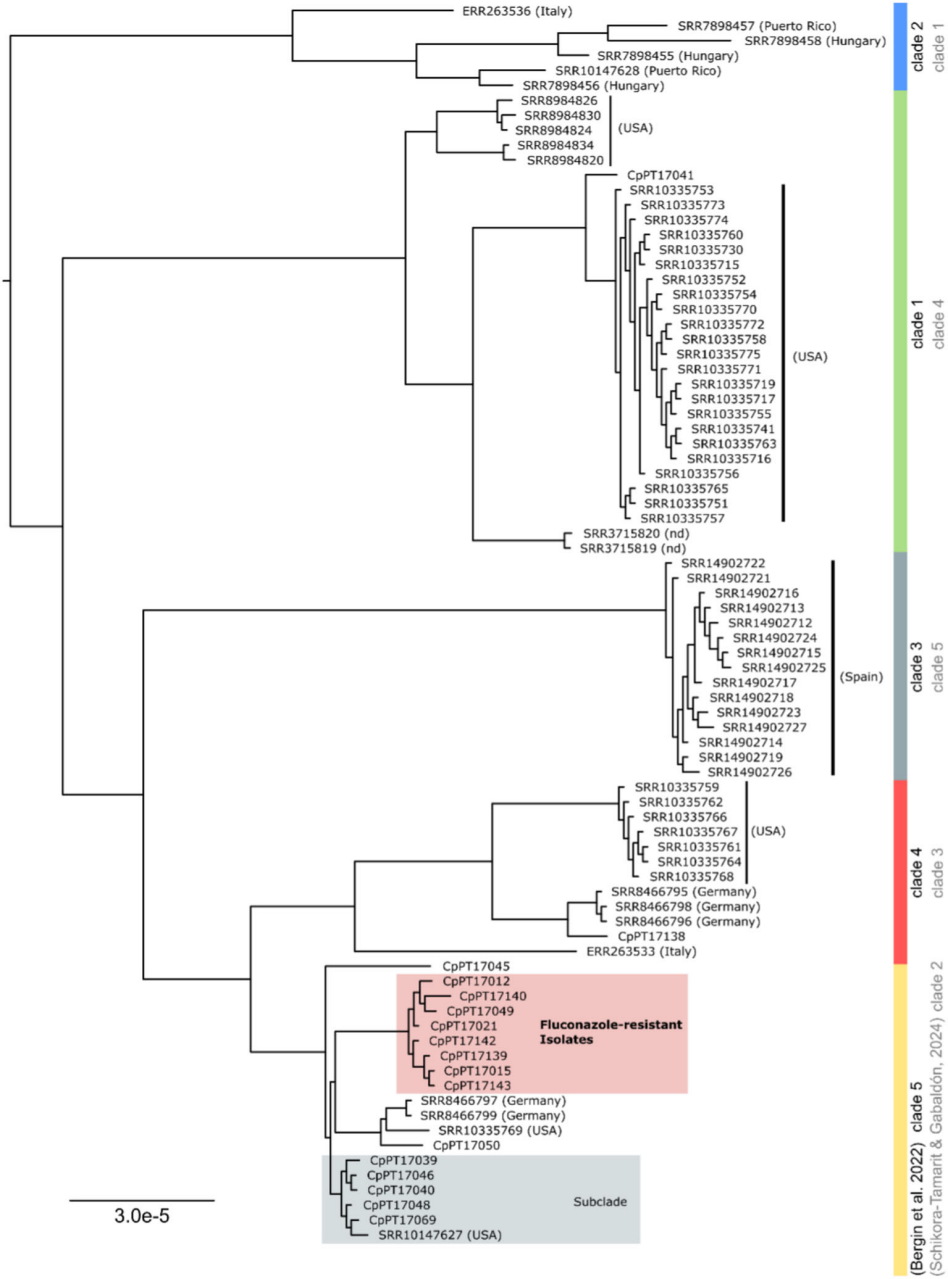
Phylogenetic characterization of the *C. parapsilosis* isolates sequenced in this study. Maximum likelihood tree reconstruction of the 12,954,955 bp alignment with 8394 variable sites, including the isolates from this study and the 66 *C. parapsilosis* isolates from the public database. The countries of origin of each isolate are indicated in brackets; “nd” - no data. Clade 1 is highlighted in green, clade 2 in blue, clade 3 in grey, clade 4 in red and clade 5 in yellow. The tree was rooted using the midpoint rooting method.

Recent genomic analysis of *C. parapsilosis* revealed the existence of at least four *C. parapsilosis* genetic clades (Bergin et al., 2022; Schikora-Tamarit and Gabaldón, 2024). Aiming to integrate the isolates sequenced in this study in the previously described *C. parapsilosis* genetic diversity and with isolates from different geographical locations, we reconstructed the phylogeny of a multi-sequence alignment comprising the 17 isolates sequenced in this study and 66 for which WGS data was available in public databases (**Supplementary File 2**, details in Material and methods section). The two previous studies describing the genetic clades of *C. parapsilosis* attribute different nomenclature (numbers) to the same clade (Bergin et al., 2022; Schikora-Tamarit and Gabaldón, 2024). Still, as they partially rely on the same set of isolates, we could infer the clade name correspondence between the two. In order to ensure backward compatibility between this study and the previous ones, we here opted to use the cluster nomenclature of Bergin et al. (2022), and present the clade name correspondence between the two studies in **Figure 3** and in **Supplementary Files 1 and 2**. Our analysis revealed that most of our isolates (N = 15) belong to *C. parapsilosis* clade 5, and only one isolate belongs to clade 4 (isolate CpPT17138) and another one to clade 1 (isolate CpPT17041) (**Figure 3**). Of note, isolate CpPT17069 and other four with a similar genetic profile and source (nail) formed a separate subcluster within clade 5, which also comprises an isolate from the USA collected from orbital tissue. Moreover, the eight fluconazole-resistant isolates identified in this study clustered together in the tree, forming a single clonal cluster within clade 5, which is consistent with the similar genetic patterns identified in these isolates (**Table 2**; **Figure 2**). Interestingly, our results do not point to an association between genetic clade and geographical location, with isolates from different continents appearing to be genetically related (**Figure 3**). Contrary to our expectations, none of the isolates from our study clustered together with the isolates from the neighbor country, Spain, which belong to clade 3.

## 4 Discussion

This study addresses a significant knowledge gap concerning the epidemiological and genomic data on fluconazole resistance patterns of *C. parapsilosis* in Portugal, which is of special importance due to the recent emergence of antifungal resistance in this species across the globe and particularly in Southern Europe (Arendrup et al., 2023). The isolates from this study were categorized based on their biological or environmental origin, and it was observed that 46% of the 145 studied isolates originated from superficial biological samples, emphasizing the frequent presence of these species on skin and nails. In addition to these, 25 isolates (17%) were sourced from a hospital or natural environments, underscoring the significance of environmental factors in facilitating the transmission of this yeast between patients, particularly through exogenous transmission (colonized environments, objects, and individuals capable of transmitting these strains to the patient) (Sabino et al., 2015).

Phenotypic assays showed that 5.5% of these isolates had elevated MIC values for fluconazole. These isolates were predominantly collected from deep infections, and none were collected from the environment, in accordance with what was observed in other studies (Díaz-García et al., 2022, 2023; Daneshnia et al., 2023; Hubler et al., 2023). All isolates collected in the period 2003–2007 were susceptible to fluconazole, thereby corroborating the study by Costa-de-Oliveira et al. (2008), which did not find any fluconazole*-*resistant *C. parapsilosis* from bloodstream infections in the Portuguese isolates studied by the authors in a similar period. A posterior study published in 2014 by Faria-Ramos et al. analyzed 55 *C. parapsilosis* isolates from bloodstream infections in Portugal, and 3.63% of those showed resistance to fluconazole, thus showing an increase in the resistance frequency from 2008 to 2014 (Faria-Ramos et al., 2014). Later on, Nascimento et al. (2024) found that, of 89 *C. parapsilosis* complex isolates collected between 2020 and 2022, 2.2% were resistant to fluconazole. Regarding the isolates included in the present study, there was a significant difference between 2003–2007 and 2017-2024, since in the first period there were no resistant isolates, whereas in the second period eight fluconazole-resistant isolates were detected, and most of these were from after 2022. Díaz-Garcia et al. (2022) analyzed 354 Spanish *C. parapsilosis* isolates from blood cultures and intra-abdominal samples, from different hospitals in Madrid metropolitan area between 2019-2021, obtaining 13.6% of resistant isolates. Despite the smaller sample size in our study, there is no significant difference in the rates of fluconazole resistance in *C. parapsilosis* between similar periods in the two studies, and both countries have a potentially worrying prevalence of resistance.

From a genetic point of view, the main mutation expected in *C. parapsilosis* fluconazole-resistant isolates was the Y132F substitution in *ERG11*, not only due to its spread in Spain and our close geographical location (Díaz-García et al., 2022), but also because this has been pointed as the main cause of azole resistance in this species (Castanheira et al., 2020; Hubler et al., 2023). Indeed, the analysis of the DNA sequences of the isolates characterized in this study as resistant to fluconazole showed that all of them had this mutation (**Table 1**), thus justifying the observed phenotype. Additionally, all of them harbored non-synonymous mutations in other genes often implicated in antifungal resistance. However, such variants should be interpreted with caution, particularly in genes where resistance is typically mediated through transcriptional overexpression rather than coding-sequence alterations. Moreover, the limited number of genomes restricts the strength of all genomic comparisons, particularly across different resistance groups. As such, their potential contribution to fluconazole resistance or to other unexplored phenotypic traits cannot be inferred without further investigation. For instance, three mutations detected in *FKS1*, *MRR1*, and *CDR1* (R871G and D877N – *FKS1*; D615G – *MRR1*; Q935K – *CDR1*) have not been previously described in the literature (**Table 2**), and, although the I396V detected in *MDR1* has already been found in other strains of *C. parapsilosis* and may be related to the overexpression of this gene, its function has not been fully described (Tóth et al., 2019; Castanheira et al., 2020; Asadzadeh et al., 2021). Without deep phenotypic studies combined with gene expression assessments, we cannot rule out the involvement of other resistance mechanisms, such as the overexpression of the *MDR1* efflux system or of *CDR1*, alone or together, with mutations in *ERG11*.

Regarding the isolates characterized as being at the threshold of susceptibility (MIC = 4 mg/L) (isolates CpPT17069 and CpPT17138), they integrate distinct genetic clades, being not closely related (**Figure 3**). While isolate CpPT17069 comprised the R398I mutation in *ERG11*, which has been previously detected in both fluconazole-susceptible and fluconazole-resistant isolates (Berkow et al., 2015; Alcoceba et al., 2022), isolate CpPT17138 did not present a non-synonymous mutation in any of genes inspected in this study, suggesting that the intermediate phenotype in the studied isolates might be, at least in the case of the latter, associated to a genetic mechanism that is not possible to determine based on our approach.

The WGS analysis also revealed the existence of multiple gene CNVs and structural variation in the Portuguese *C. parapsilosis* isolates analyzed (**Figure 2**). These results are aligned with previous studies showing that CNVs are an important way for *C. parapsilosis* to acquire genetic diversity, and, particularly, that genes like *RTA3* (CPAR2_104610) and *ARR3* (CPAR2_601050) are under significant selective pressure (Zhai et al., 2020; Bergin et al., 2022; Selmecki, 2023). Noteworthy, although we did not see an association between these events and the fluconazole-resistance phenotype, it is worth noting that they can lead to resistance to other antifungals, such as miltefosine, a drug which usage for the treatment of invasive candidiasis was recently approved by the United States Food and Drug Administration (FDA) (Bergin et al., 2022) (https://www.accessdata.fda.gov/scripts/opdlisting/oopd/detailedIndex.cfm?cfgridkey=843921). Importantly, the functional consequences of the observed CNVs remain to be experimentally validated, and their phenotypic relevance should therefore be interpreted cautiously.

Despite the small number of samples analyzed by WGS in this study, we found three distinct *C. parapsilosis* genetic clades in the Portuguese isolates analyzed, showcasing a broad diversity of isolates. Contrary to our initial expectations, our isolates were found to be more genetically similar to isolates from the USA and Germany than to those from Spain, despite Spain being the geographically closest country. This suggests that the proximity of centers of dissemination for these pathogens is not necessarily indicative of genomic similarity, as genetic patterns specific to each location may exist. Most of the isolates of the dataset of this study belong to clade 5 and, interestingly, were divided into two main clonal lineages (**Figure 3**). One of them corresponds mainly to the isolates from superficial infections, more specifically nails, suggesting a potential tropism of this genetic background. The other clonal lineage within clade 5 included all fluconazole-resistant isolates detected within our dataset, which suggests that the increased fluconazole resistance in *C. parapsilosis* that has been observed in the last years might be associated with the introduction or emergence of this lineage in the country. Nevertheless, since the majority of our isolates came from the Lisbon metropolitan area, we cannot discard the existence of a geographic bias in our results. Still, since the majority of those resistant isolates (six of them) came from a single hospital and were collected in different years, we cannot rule out the existence of a local persistent clonal lineage. Future studies not only comprising larger datasets and integrating epidemiological, phenotypic and genomic data but also covering a vast panoply of antifungals, will be essential to get a better understanding of the population genomics of *C. parapsilosis* and clarify the proposed scenario.

## 5 Conclusions

This study has made a significant contribution to the advancement of knowledge in the field of fluconazole-resistant *C. parapsilosis* in the largest collection of Portuguese isolates published to date. Future studies must continue this line of research, with a particular focus on the genomic differences found in isolates from different origins, as well as the various deletion events of certain genes. This is particularly important given that many of the deletions found are in genes whose function in *C. parapsilosis* is unknown. Consequently, these findings must be thoroughly investigated in both *in vitro* and *in vivo* settings to elucidate their implications for fitness or resistance to fluconazole and other antifungals. This comprehensive approach will facilitate the identification of novel therapeutic targets, thereby expanding the scope of related studies. As *C. parapsilosis* demonstrates considerable phenotypic plasticity, as it is present in humans, animals, and in natural environments (Brilhante et al., 2019; World Health Organization, 2022), the emergence of resistance must be addressed within the context of the One Health concept, given the global utilization of antifungals in agriculture, industry, and living beings.

## Data availability statement

Whole-genome sequencing data generated in this study are available at the European Read Archive (ENA) under BioProject PRJEB64175.

## Supplementary material

Supplementary file 1 List of the 145 *C. parapsilosis* isolates screened in this study and respective metadata. Results of antifungal susceptibility tests are also presented, as well as information on the WGS-based clade attribution.

Supplementary file 2 List of the 66 *C. parapsilosis* isolates retrieved from public databases and respective metadata. Information on the WGS-based clade attribution is also provided.
